# Teachers’ Beliefs and Practices Regarding the Role of Executive Functions in Reading and Arithmetic

**DOI:** 10.3389/fpsyg.2016.01567

**Published:** 2016-10-17

**Authors:** Shirley Rapoport, Orly Rubinsten, Tami Katzir

**Affiliations:** The Edmond J. Safra Brain Research Centre for the Study of Learning Disabilities, Department of Learning Disabilities and Special Education, University of HaifaHaifa, Israel

**Keywords:** pedagogical practices, executive functions, reading, arithmetic

## Abstract

The current study investigated early elementary school teachers’ beliefs and practices regarding the role of Executive Functions (EFs) in reading and arithmetic. A new research questionnaire was developed and judged by professionals in the academia and the field. Reponses were obtained from 144 teachers from Israel. Factor analysis divided the questionnaire into three valid and reliable subscales, reflecting (1) beliefs regarding the contribution of EFs to reading and arithmetic, (2) pedagogical practices, and (3) a connection between the cognitive mechanisms of reading and arithmetic. Findings indicate that teachers believe EFs affect students’ performance in reading and arithmetic. These beliefs were also correlated with pedagogical practices. Additionally, special education teachers’ scored higher on the different subscales compared to general education teachers. These findings shed light on the way teachers perceive the cognitive foundations of reading and arithmetic and indicate to which extent these perceptions guide their teaching practices.

## Introduction

“Executive functions” (EF) are typically defined as “general-purpose control mechanisms that modulate the operation of various cognitive sub-processes and thereby regulate the dynamics of human cognition” (Miyake et al., 2000; Miyake and Friedman, 2012). They allow individuals to be goal-directed and adaptively select their responses, rather than respond in an automatic fashion (Garon et al., 2008) by relying on instinct or intuition (Diamond, 2013). Core EF components, such as inhibition, shifting and working memory begin to develop during infancy (Garon et al., 2008), predicting school readiness in the language and social-emotional domains (Bierman et al., 2008). EF continue to develop into adulthood, forming the foundation for higher cognitive processes (Garon et al., 2008) affecting mental and physical health, job success, intimate relationships and social behavior (Diamond, 2013).

In recent years, there is also accumulated research regarding the contribution of domain-general EF to the development of reading and arithmetic (e.g., Durand et al., 2005; Altemeier et al., 2008; Cartwright, 2012; Compton et al., 2012; Ashkenazi et al., 2013; Georgiou et al., 2013; Miller et al., 2013; Davidse et al., 2015). The domains of reading and arithmetic have been traditionally linked to separable underlying cognitive mechanisms. Reading is considered to be dependent on linguistic processes (Lyon et al., 2003) while arithmetic depends on abstract manipulation of quantities (Price et al., 2007). Yet, first of all, semantic and arithmetical concepts are both communicated through formally acquired symbol systems. Alphabetic writing systems and Arabic numerals even emerged from the same source – counting coins, which are tracked back to 8000BC (Wolf, 2008).

Furthermore, apart from the shared origin of symbol systems, reading and arithmetic depend on shared cognitive mechanisms. Most of these mechanisms, as mentioned, can be conceptualized as domain-general EF (amongst other cognitive mechanisms, such as general verbal ability, e.g., Krajewski and Schneider, 2009; Schroeder, 2011; Bar-Kochva, 2012; Vukovic and Lesaux, 2012). For example, EF in preschool accounted for substantial variability in mathematical and reading achievement 2 years later (Röthlisberger et al., 2013). In another study, neuropsychological tests and teacher reports of EFs accounted for as far as 40% of variance in English scores and 30% of variance in mathematics scores in the 4th grade (Waber et al., 2006).

With all that being said, it is still unclear whether this important link between EF and school achievements has any expression in pedagogical practices. As much as this link is well established in scientific literature, we have yet to understand how it is reflected in teachers’ beliefs, as well as in their practices (Pajares, 1992). For example, if a teacher witnesses some students struggle with all schoolwork, is he/she exposed to the possibility that these challenges may stem from an underlying difficulty in executive functioning?

To discover whether recent scientific findings are bridged into pedagogy, the current study will look into elementary school teachers’ beliefs regarding the link between EF and achievements in reading and arithmetic. We will focus on teachers’ beliefs, due to the crucial influence they may exert on pedagogical practices (Borg, 2001; Baccus, 2004; Cross, 2009), and learning opportunities (Falcón-Huertas, 2006; Kaya, 2014). We will also explore to what extent teachers’ beliefs correlate with their reported pedagogical practice in arithmetic and reading instruction, and examine patterns of beliefs and practices in different populations of teachers (differing by level of experience and type of students taught).

Past studies of teachers’ beliefs have typically focused on teachers’ attitudes toward themselves (Tschannen-Moran and Hoy, 2007; van Uden et al., 2014), their students (Lavigne, 2014) and the nature of teaching and learning (Windschitl and Sahl, 2002; Kim et al., 2013). Other studies examined teachers of specific academic domains, focusing, for example, on attitudes toward literacy (Martin et al., 2007; Jiménez et al., 2015; Ritchey et al., 2015), such as stressing phonics as opposed to a whole-language approach when teaching reading (DeFord, 1979). Other studies explored beliefs regarding mathematics instruction (Lerman, 1990; Stipek et al., 2001; Sweeting, 2011), for example, stressing answer correctness as opposed to focusing on understanding mathematical concepts (Stipek et al., 2001). However, to our knowledge, there is no published study that focuses on teachers’ beliefs about the connection between EF and school achievements in reading and arithmetic. Thus, the current study addresses an important gap in the literature, by exploring teachers’ beliefs and practices regarding the role of domain-general cognitive mechanisms such as EF on domain-specific subject matters such as reading and arithmetic.

Before describing research questions and methods, scientific literature regarding the contribution of EF to both reading and arithmetic will be reviewed, along with literature about the connection between the two domains. Lastly, the connection between teachers’ beliefs and their pedagogical practices will be reviewed.

### Conceptualization of Executive Functions in the Current Study

Different researchers included different cognitive mechanisms under the umbrella of EF (e.g., compare Jurado and Rosselli, 2007 with Barkley, 1997). Furthermore, some studies refer to “EFs” as one general construct (e.g., Waber et al., 2006; Best et al., 2011; Röthlisberger et al., 2013), while others study the contribution of the different EFs separately (e.g., Inhibition and working memory, Borella et al., 2010; Planning, updating in working memory and inhibition, Kroesbergen et al., 2009; Inhibition, shifting and updating, Van der Ven et al., 2012). Both approaches have merit, as EFs differ in terms of both cognition and biology (e.g., Miyake et al., 2000; Gunning-Dixon and Raz, 2003; Jurado and Rosselli, 2007), yet share some underlying commonalities (Miyake et al., 2000). In addition, EF sub-processes work in conjunction many times (Alvarez and Emory, 2006) and it might be hard to separate their joint contribution to academic achievements.

Since the incorporation of teaching methods addressing EF in the school curriculum is relatively new and still not widespread (Dias and Seabra, 2015), studies linking specific EF to the acquisition of both reading and arithmetic will be reviewed, but when addressing teachers’ beliefs in the current study, the different EFs and EF-related mechanisms reviewed will be later conceptualized as one joint construct.

## The Contribution of Different Executive Functions to Reading and Arithmetic

Some specific EF and EF-related mechanisms that have been shown to predict reading and arithmetic are: Inhibition (e.g., Altemeier et al., 2008; Toll et al., 2011), attentional control (Welsh et al., 2010), cognitive flexibility or shifting (e.g., Altemeier et al., 2008; Yeniad et al., 2013), planning (e.g., Sesma et al., 2009), working memory (e.g., McVay and Kane, 2012; Miller et al., 2013) and fluent retrieval of information from long-term memory (e.g., Ashkenazi et al., 2013; Georgiou et al., 2013).

### EF and Reading

Inhibitory control (i.e., the ability to restrain responses; Blair and Razza, 2007), and attention control (the ability to control the focus on particular information; Welsh et al., 2010) were found to have a significant relationship with pre-reading skills in kindergarten. In elementary school, inhibition (Altemeier et al., 2008) and shifting (i.e., changing the mental set that has been learned to a new one; Yeniad et al., 2013) were linked to general reading performance (Yeniad et al., 2013) and particular decoding and word-reading measures (van der Sluis et al., 2007; Altemeier et al., 2008).

Executive function also plays a role in reading comprehension: attention shifting and inhibitory control was uniquely associated with reading comprehension in the 4th grade, controlling for working memory, processing speed and phonological awareness (Kieffer et al., 2013). Along the same lines, poor comprehenders in elementary and middle school were shown to lack inhibition (De Beni and Palladino, 2000) and also planning (deciding which tasks are necessary to complete a goal, and in what order; Sesma et al., 2009) abilities, compared to good comprehenders.

### EF and Math

In the field of arithmetic, attention control (Welsh et al., 2010) and planning (Kroesbergen et al., 2009) predicted emergent numeracy skills. More EFs found to predict arithmetical ability are inhibition, underlying factual and procedural knowledge, and shifting, underlying procedural and conceptual arithmetical knowledge (Cragg and Gilmore, 2014). Inhibition predicted early school math achievement (Kroesbergen et al., 2009; Clark et al., 2010; Gilmore et al., 2013; Viterbori et al., 2015) and discriminated children with mathematical difficulties from typically achieving children in 1st and 2nd grade (Toll et al., 2011). Students in 3rd–5th grades with weak inhibition skills mixed conceptual knowledge with an incompatible computational algorithm, suggesting they had the right knowledge but failed to inhibit a previously well-learned algorithm (Robinson and Dubé, 2013).

Shifting ability is also generally thought to predict performance in mathematics (Yeniad et al., 2013). It predicted early school math achievement (Clark et al., 2010) and was correlated with arithmetic abilities in children aged 9–12 (van der Sluis et al., 2007). However, these findings are debatable since some studies did not find inhibition and shifting abilities to predict math achievement in early elementary school (e.g., Van der Ven et al., 2012).

## Contribution of EF-Related Cognitive Mechanisms to Reading and Arithmetic

Along with cognitive mechanisms defined as EF *per se*, there are additional cognitive mechanisms related to EF, which are significantly related to the development of reading and arithmetic.

Working memory (Baddeley, 1992) has been closely linked to executive functioning. For example, McCabe et al. (2010), have found a correlation of 0.97 between the constructs of WM capacity and EF. WM has been found to contribute greatly to academic performance in reading and arithmetic from preschool to older children (e.g., Alloway et al., 2005; Berg, 2008; Krajewski and Schneider, 2009; Geary, 2011). It has been shown to be a crucial contributor to literacy and numeracy skills in preschool and later on (Alloway and Alloway, 2010; Miller et al., 2013).

WM strongly predicted math achievement in 1st–3rd grade (Toll et al., 2011; Viterbori et al., 2015). In particular, WM is considered to underlie both factual and procedural arithmetical knowledge (Cragg and Gilmore, 2014). One possible explanation for these ties are that calculation seems to rely on WM processes, since it involves storing temporary information while performing a mental operation on it, especially when the problem is presented verbally rather than visually (Berg, 2008). In addition, WM was found to contribute to strategy implementation in solving math problems (Geary et al., 2004; Lemaire, 2010).

In reading, Swanson et al. (2009) claimed that WM deficits contribute to problems in learning to read, supported by Nevo and Breznitz’s (2011) findings that measures of verbal WM predict decoding and reading rate. Conversely, other studies of early literacy development show that verbal short-term memory but not WM *per se*, is related to word decoding proficiency, especially in primary grades (Alloway et al., 2005). At later stages of reading development, WM predicted reading comprehension (Berninger et al., 2010; Geary, 2011; Bar-Kochva, 2012; McVay and Kane, 2012).

Retrieval from long-term memory is another domain-general cognitive mechanism strongly tied to executive functioning, as fact retrieval employs bidirectional hippocampal-prefrontal connections (Cho et al., 2012) and is affected by working memory span (Rosen and Engle, 1997; Unsworth et al., 2013) and attentional processes (Kane and Engle, 2000). Some even conceptualize verbal fluency as an EF (Jurado and Rosselli, 2007).

It is known that retrieval underlies both reading and arithmetic (Kulak, 1993; Koponen et al., 2007). In both domains, the sequence of skill development involves a shift from time-consuming procedural strategies – effortful phonemic analysis in reading and counting in arithmetic – to automatic retrieval of high frequency words/arithmetic facts, which enables the learner to devote resources to “higher” tasks like reading comprehension or solving mathematical word problems (Kulak, 1993). It has been also claimed that flawed retrieval can cause a learning difficulty related to reading and arithmetic (Ashkenazi et al., 2013), as both domains rely on the fast retrieval of phonological information from long-term memory (Georgiou et al., 2013). Evidence also shows that retrieval is a main problem for children with dyslexia (Hanly and Vandenberg, 2010) and children with mathematical difficulties (Geary, 2004).

## Overall Relationships Between Reading and Arithmetic Development

All findings described above show clearly that shared domain-general mechanisms greatly contribute to performance in both reading and arithmetic. Considering shared underlying mechanisms, it is not surprising that for many years there are consistent findings demonstrating that that gains in reading abilities positively affects arithmetic skills dates (e.g., Gilmary, 1967). Further, recent studies suggest that dyslexics experience in difficulties in calculations (Miles and Miles, 1992; Mammarella et al., 2013). Early reading skills were found to be important for success in math, as reading comprehension in the 3rd grade predicting arithmetic skills in 3rd–8th grades (Grimm, 2008). A more recent study found a positive correlation between growth rates in reading and mathematics abilities throughout 4th–7th grades (Shin et al., 2013). In compliance with Grimm (2008), this correlation was also attributed by the authors to the influence of growth in reading ability on growth in mathematics (Shin et al., 2013).

There are some exceptions to this hypothesis regarding a single-directed influence of reading on arithmetic. For example, a Finnish longitudinal study of 1st and 2nd graders found an association between reading comprehension and mathematical abilities, while mathematical abilities, surprisingly, predicted subsequent reading comprehension rather than vice versa (Lerkkanen et al., 2005). Nevertheless, in general, reading performance seems to positively affect math performance but not vice versa (Jordan et al., 2002; Near, 2014).

To summarize, EF, separately or as a joint construct, have been strongly linked to school achievements in both reading and arithmetic (e.g., Altemeier et al., 2008; Cartwright, 2012; Compton et al., 2012; Ashkenazi et al., 2013; Georgiou et al., 2013; Miller et al., 2013; Davidse et al., 2015). In line with these findings, a correlation emerges between student achievements in reading and achievements in arithmetic (e.g., Grimm, 2008; Shin et al., 2013; Near, 2014).

In light of these strong links, the current study wishes to explore teachers’ beliefs and practices regarding EF and their role in learning reading and arithmetic. The importance of teachers’ beliefs to their pedagogical practices will be reviewed in the following paragraphs. Afterward, teacher variables which might influence teachers’ beliefs are discussed.

## Teaching Beliefs and their Connection to Pedagogical Practices

A belief, in general, is a proposition held and accepted by an individual as true (Borg, 2001). Beliefs evoke emotional obligation in the individual and guide him/her in their thoughts and behavioral practices (Borg, 2001). Thus, teaching, or pedagogical, beliefs are an individual’s beliefs relevant to their teaching abilities, the role of a teacher, the nature of learning etc. (Borg, 2001).

Teachers hold a variety of beliefs about the nature of their field of teaching, the way it should be taught and learned, their teaching ability etc. (e.g., Luciano, 1997; Westwood et al., 1997; Buehl et al., 2002; Baccus, 2004; Cross, 2009). These beliefs hold great importance, since reforms in the curriculum depend on the ability of policy makers to change teachers’ beliefs about the way children learn (Lloyd, 2003). It has also been claimed that “attention to teachers’ beliefs can inform educational practice in ways that prevailing research agendas have not and cannot” (Pajares, 1992).

Such claims point to the direct relation between teaching beliefs and pedagogical practices, which are the ways teachers choose to transfer knowledge to their students in the classroom. This connection was found as early on as kindergarten, where educational beliefs of teachers predicted children’s learning opportunities above teacher’s education and experience (Paro et al., 2009). In the field of reading instruction, a relationship was found between 1st grade teachers’ theoretical orientation toward reading, ranging from phonics instruction to “whole-language” instruction (TORP questionnaire; DeFord, 1979) and their instructional practices (Luciano, 1997). Another study found an association between teachers’ beliefs and the amount of instructional time spent on different aspects of reading instruction (Baccus, 2004). In mathematics, similarly, beliefs of 4th–6th grade teachers correlated with their classroom practices (Stipek et al., 2001) and another qualitative study found an association between beliefs of 9th grade algebra teachers and teaching practices (Cross, 2009).

Evidence showed that teaching practices are associated not only with beliefs, but they were also linked to students’ conceptions. Students’ conceptions regarding the nature and purpose of reading were affected by their teachers’ literacy beliefs and practices (Falcón-Huertas, 2006). Another research found that teachers’ beliefs about the importance of children’s literature in reading instruction, affected positively their students’ reading practices (Kaya, 2014). In the field of mathematics, a correlation was found between math teachers’ beliefs and teaching practices and students’ beliefs about mathematics (Carter and Norwood, 1997).

Naturally, teaching beliefs are not always aligned with teachers’ pedagogical practices. Liu (2011), for example, found that even though most Taiwanese teachers held learner-centered beliefs, most classroom activities, when using technology, were still lecture-based rather than learner-based. Teaching practices are sometimes affected by “classroom realities” (Ertmer et al., 2012). Even though, it is important to note that personal beliefs are still the most influential factor on pedagogical practices (Ertmer et al., 2006), and it has been claimed that a change in practices can be achieved only if teachers’ attitudes and beliefs are addressed (Ertmer et al., 2012).

## Beliefs, Practices and Teacher Variables

It seems likely that different professional variables may have an effect on teachers’ beliefs and practices regarding the effect of EF on learning. Special education teachers may give more weight to the role of EF in the development of literacy and mathematics. First of all, they usually have more in-service training about handling students with ADHD (Martinussen et al., 2011; McKnight, 2015), a difficulty in executive functioning (Barkley, 1997). Previous research has also indicated that special education teachers report a greater executive functioning difficulty in their students, compared to general education students (Wright, 2010). Meltzer et al. (2007) have even claimed that interventions addressing EF would result in less special-education referrals. Hence, it is likely that the special education teacher, who is both more trained and more regularly exposed to EF difficulties in the classroom, would display more beliefs and teaching practices to address the connection of EF to school achievements.

Teaching experience could also affect teachers’ outlook on the connection between EF and school achievements. Previous research has shown that teaching experience is linked to a greater sense of teacher efficacy: More experienced teachers are more confident in their professional ability (Wolters and Daugherty, 2007; Rubie-Davies et al., 2012). Other distinctions between novice and experienced teachers were drawn in other fields, such as pedagogical knowledge (Silberstein and Tamir, 1991), problem solving (Swanson et al., 1990) and decision making (Housner and Griffey, 1985). There is some indication of a possible link between beliefs about EF and teaching experience. Experienced teachers were found to possess higher knowledge of characteristics of and treatments for ADHD than inexperienced ones (Anderson et al., 2012). ADHD, as mentioned, is manifested in difficulties in executive functioning (Barkley, 1997), and these results may suggest that as teachers become more experienced, they understand more about the manifestation of EF in the classroom. Consequently, it seems possible they hold more beliefs and practices concerning the effect EF have on all student achievements.

## The Current Study

The main aim of this study was to add to the scarce literature on teachers’ beliefs about the importance of EF. It introduces a novel focus on early elementary teachers’ beliefs and their correlation with reported classroom practices, regarding EF in reading and arithmetic classes, considering the heavily reported effect of EF on school achievements in reading and arithmetic as early on as kindergarten (e.g., Best et al., 2011; Miller et al., 2013). The current study explored to what extent, in teachers’ beliefs, achievements in reading were correlated with achievements in arithmetic and vice versa (e.g., Grimm, 2008; Shin et al., 2013). Different patterns of beliefs and practices in different populations of teachers (differing by level of experience and type of students taught) were being examined. The study was approved by Haifa University’s IRB ethics committee.

To achieve these aims, we developed and validated a novel research questionnaire, containing statements tapping teaching beliefs about the contribution of EF to achievements in reading and arithmetic, practices targeting this contribution and the connection teachers perceive between students’ achievements in reading and arithmetic. Data was cottlected from a large pool of obtaining early elementary school teachers (of 1st–4th grades).

Findings from the questionnaire were subject to factor analysis and differences between groups analysis.

Using our novel questionnaire, we wished to discover the relationship between teaching beliefs and practices regarding the contribution of EF to academic achievement, amongst early elementary school teachers of reading and arithmetic. We also wanted to characterize the relationship between teaching experience and those beliefs and practices, as well as the relationship between teaching experience and the perception of a connection between achievements in reading and arithmetic. Furthermore, we wanted to examine whether elementary school teachers in general and special education differ in their beliefs and practices regarding EF. Hence, such a questionnaire may facilitate and focus teachers’ attention to their own understanding of EF, their role in learning and development, and how they can support its development.

We hypothesized that a positive correlation will be found between teaching beliefs and practices regarding the contribution of EF to academic achievement. We further hypothesized that a positive correlation will be found between teaching experience and those beliefs and practices, along with a stronger perception of a connection between reading and arithmetic achievements. Lastly, we hypothesized that special education teachers, compared to general education teachers, will hold more beliefs about the contribution of EF to academic achievements in both reading and arithmetic class (e.g., McKnight, 2015), apply more teaching practices targeting EF in their classes and see a stronger connection between achievements in reading and arithmetic.

## Materials and Methods

### The Sample

The sample was comprised of 144 respondents. The questionnaire was sent as an online survey or handed in a hard-copy version. The sampling procedure was generally a convenience sample, as respondents were recruited through personal acquaintances and social networks (such as Facebook). Another sampling practice was to recruit teachers attending professional development courses and students in teacher-training programs. Questionnaire responses were collected in five different professional development programs, one of them national and four of them regional courses, half of them held in the northern Haifa district and half in the central Sharon district. Hundred and sixteen participants, who responded to more than 80% of the questionnaire, were included in the statistical analysis (see **Table 1** for demographic characteristics of the sample).

**Table 1:** Individual characteristics of teachers in the study.

Characteristic	Sample (*n* = 116)
Education system	
General	61.21% (71)
Special	38.79% (45)
Teacher’s academic education	
B.A.	70.69% (82)
M.A.	29.31% (34)
Certified teacher?	
Yes	74.14% (86)
Student	25.86% (30)
Level of experience teaching reading	
None/Less than 1 year	19.83% (23)
1–5 years	31.90% (37)
Over 5 years	48.28% (56)
Level of experience teaching arithmetic	
None/Less than 1 year	26.72% (31)
1–5 years	31.90% (37)
Over 5 years	41.38% (48)
Homeroom/specialized teacher	
Do not currently teach	17.24% (20)
Homeroom	58.62% (68)
specialized teacher	24.14% (28)
Grades taught by teacher	
Do not currently teach	17.24% (20)
1st–2nd	35.34% (41)
3rd–4th	16.38% (19)
1st–4th	31.03% (36)

### Survey Instrument

A new pilot questionnaire was composed for the purpose of the current study. In the development stage, the conceptual framework was constructed based on EF components identified in the literature. The preliminary questionnaire was divided into two sections. The first section consisted of 15 items inquiring about demographic characteristics of the respondent (See Appendix 1). The second section consisted of a large pool of 69 items, reflecting 10 theoretical themes (See Appendix 2). Seven themes regarded the connection between reading and arithmetic abilities and the following EFs: automatic retrieval, working memory, planning, shifting (cognitive flexibility), inhibition and attentional control. An additional group of items addressed the beliefs about the need to explicitly teach EF-enhancing strategies at school. Two other themes targeted the connection between academic abilities and reading: general verbal ability and phonological awareness. The last group of items tapped the perceived connection between achievements in reading and arithmetic – are they based on shared mechanisms? The different theoretical themes contained questions about theoretical beliefs and teaching practices. In the pilot phase, the questionnaire was reviewed for relevance, simplicity, clarity and ambiguity by six professionals in the relevant academic field and a group of ten representatives of the relevant population (professional early elementary school teachers), in order to obtain content validity (Yaghmale, 2003).

Items reflecting 7 of the 10 original theoretical scales were included in the analysis, considering the sample size. In order to perform dimension reduction of the data, a completion of missing values and item pruning were first administered. First of all, items with a response rate lower than 80% were not included in the analysis, as well as respondents who answered less than 80% of the items. The remaining missing values were completed by the median of responses to the same item. Due to statistical redundancy, if two or more items were highly correlated (*R* > 0.5), only one of them, chosen according to theoretical considerations, was included in the analysis. Items uncorrelated (*R* < 0.2) with other items which reflect the same theoretical theme, were also not included.

Exploratory factor analyses using maximum likelihood (ML) factoring, followed by direct oblimin rotation, were then administered. The ML factor extraction method was chosen due to normal distribution of the data (distribution of responses to all items met the criteria of skewness <2, kurtosis <7). The direct oblimin rotation, an oblique rotation, was chosen since these rotations can produce a structure with correlated factors, as opposed to orthogonal rotations (such as principal axis), which do not permit correlations among factors (Fabrigar et al., 1999; Costello and Osborne, 2005). It was defined that only items with factor loading of over 0.4 on only one of the factors, were to be included.

### Description of Principal Factors

Twenty two items were grouped by the factoring procedure into three factors. Factor score was calculated using the regression method. All three factor scores were normally distributed (skewness <2, kurtosis <7). Each item was given an identifying code, in order to simplify statistical analysis and chart display (see **Table 2**).

**Table 2:** Factor loadings with direct oblimin rotation of final questionnaire items.

Item	(1) Teaching practices (TP)	(2) eading-arithmetic correlation (RAC)	(3) Teaching beliefs (TB)
I teach, in language arts class, strategies to remember in parallel multiple details from the text	0.634		
I teach in math class strategies for planning ahead in task performance	0.594		
I teach in language arts class strategies to focus on task	0.543		
When I teach a student with difficulties in reading, I will work with him on methods to store in his memory multiple bits of information in parallel	0.519		
I devote time in math class to memorizing solutions to common math problems	0.467		
When I teach a student with difficulties in math, I will work with him on methods to store in his memory multiple arithmetical operations in parallel	0.520		
I devote time in language arts class to memorizing common orthographic patterns, to encourage reading them as whole words instead of their phonological decoding	0.426		
Most of the children who read well are also good in math		-0.648	
If a student has difficulty in both reading and math, these difficulties usually stem from the same source		-0.657	
Students who do not read accurately have difficulties in understanding math		-0.613	
There are more students who have difficulties both in reading and math, than students with difficulties in math only and not in reading		-0.649	
There are more students who have difficulties both in reading and math, than students with difficulties in reading only and not in math		-0.589	
The basic mechanisms crucial for learning math are also crucial for learning to read		-0.574	
Children who can plan ahead their actions in performing a task, solve math problems more easily			0.599
The ability to focus on task is important when solving math problems			0.585
Students who are able to plan ahead their actions in performing a task, cope better with math word problems.			0.527
Students with difficulties in reading comprehension also tend to try solving problems again and again in the same way, even if this way was proven wrong			0.518
One has to keep in memory information while reading, in order to achieve reading comprehension			0.455
The ability to focus on task is important for reading comprehension			0.462
Students with difficulties in math also tend to try solving problems again and again in the same way, even if this way was proven wrong			0.474
Inhibition is an important ability in the acquirement of reading			0.455
The student’s ability to quickly recall the spelling of words he has previously been exposed to, affects reading rate			0.428

The factors extracted reflect three theoretical conceptual subscales: (1) “Teaching practices (TP),” tapping practices regarding the effect of EF on reading and arithmetic, (2) “Reading-Arithmetic connection (RAC),” tapping the perceived connection between reading and arithmetic abilities, and (3) “Teaching beliefs (TB),” tapping beliefs regarding the effect of EF on “reading and arithmetic.”

#### Subscale (1)

Teaching practices*:* contains seven items with factor loadings of 0.426–0.634. (See **Table 2**). To determine subscale reliability, internal consistency (Cronbach’s α) was tested and found to be 0.774.

#### Subscale (2)

Reading-arithmetic connection*:* contains six items with factor loadings of 0.574–0.657. (see **Table 2**). The internal consistency (Cronbach’s α) of this subscale was found to be 0.791.

#### Subscale (3)

Teaching beliefs*:* contains nine items with factor loadings of 0.428–0.599 (see **Table 2**). The internal consistency (Cronbach’s α) of this subscale was found to be 0.751.

Subscales TP and TB of the final questionnaire, tapping teaching practices and beliefs regarding the effect of EF on reading and arithmetic, include at least one item about every EF reflected in the preliminary survey instrument (see **Table 3**).

**Table 3:** The theoretical themes reflected in subscales (TP) and (TB) of the Final Questionnaire (number of items in parentheses).

Executive functions	Reading	Arithmetic
Shifting (cognitive flexibility)	Beliefs (1)	Beliefs (1)
Inhibition	Beliefs (1)	
Attentional control	Beliefs (1), Practices (1)	Beliefs (1)
Planning		Beliefs (2), Practices (1)
Working memory	Beliefs (1), Practices (2)	Practices (1)
Automatic retrieval	Beliefs (1), Practices (1)	Practices (1)

### Correlations between Extracted Factors

The factoring process used an oblique rotation, permitting the extraction of a structure with correlated factors. Indeed, such a structure was produced. Factors 1 (TP) and 3 (TB) were highly correlated (*r* = 0.512, *p* < 0.01), in compliance with research hypothesis (1). Factors 2 (RAC) and 3 (TB) were moderately correlated (*r* = 0.319, *p* < 0.01) (see **Table 4**).

**Table 4:** Correlations between extracted factors.

Measure	(1) Teaching practices (TP)	(2) Reading-arithmetic correlation (RAC)	(3) Teaching beliefs (TB)
Factor 1. TP	–		
Factor 2. RAC	0.154	–	
Factor 3. TB	0.512^∗∗^	0.319^∗∗^	–

*^∗∗^*p* < 0.01; *N* = 116.*

Correlations between subscales were also compared across research groups (general and special education teachers). Subscales 1 (TP) and 3 (TB) were correlated for both comparison groups [general education. *r*(71) = 0.518, *p* < 0.01; special education. *r*(45) = 0.471, *p* < 0.01]. Subscales 2 (RAC) and 3 (TB) were correlated for general education teachers only [*r*(71) = 0.356, *p* < 0.01]. All correlations were compared using a Fischer’s *Z* test, and no significant differences were found between these correlations.

## Statistical Analysis and Results

### Descriptive Analysis of Item Responses

Each respondent’s score on every subscale was determined as the mean of the respondent’s ratings of the statements comprising the subscale. Most respondents scored well above 3 on a likert scale on subscales 1 (TP) and 3 (TB), as reflected in the percentage of respondents who scored over 3.5. On the contrary, there was no clear trend emerging from respondents’ scores on subscale 2 (RAC). Approximately half of the respondents scored between 2.5 and 3.5. In addition, the variance of scores is high compared to the other subscales (see **Table 5**).

**Table 5:** Descriptive measures of questionnaire subscale scores.

Subscale	Min.	Max.	Mean	*SD*	% scores > 3.5	% scores < 2.5
(1) Teaching practices (TP)	1.43	4.86	3.68	0.66	62.93%	6.89%
(2) Reading-arithmetic connection (RAC)	1.67	4.83	2.88	0.81	19.83%	31.03%
(3) Teaching beliefs (TB)	2.56	5	3.97	0.53	82.76%	0%

### Analysis by Demographic Variables

Teachers were asked in the questionnaire to state the type of students they teach (general or special education) and their years of experience teaching reading and arithmetic. Both questions regarding experience were highly correlated [*r*(116) = 0.74, *p* < 0.01], suggesting statistical redundancy, thus their results were transformed into an “overall years of experience” variable.

Testing research hypothesis (2), a negative correlation was found between the average score on subscale 2 (RAC) and overall teaching experience [*r*(116) = -0.192, *p* < 0.05]. No significant correlations were found between the other subscales and overall teaching experience.

Research hypothesis (3) was tested using general linear modeling, with the average scores on the three emergent questionnaire subscales as dependent variables and the independent variable of “Education System,” based on demographic information.

To determine the connection between the education system (general/ special) the teacher belongs to and questionnaire subscale scores, multivariate analysis of covariance (MANCOVA) was conducted, with “overall years of experience” as a covariate. The latter variable was considered a confound due to the significant difference in average years of experience in the general education (*M* = 9.69, *SD* = 7.68) and special education (*M* = 4.87, *SD* = 7.72) groups; *t*(114) = 3.29, *p* < 0.01.

**FIGURE 1:**
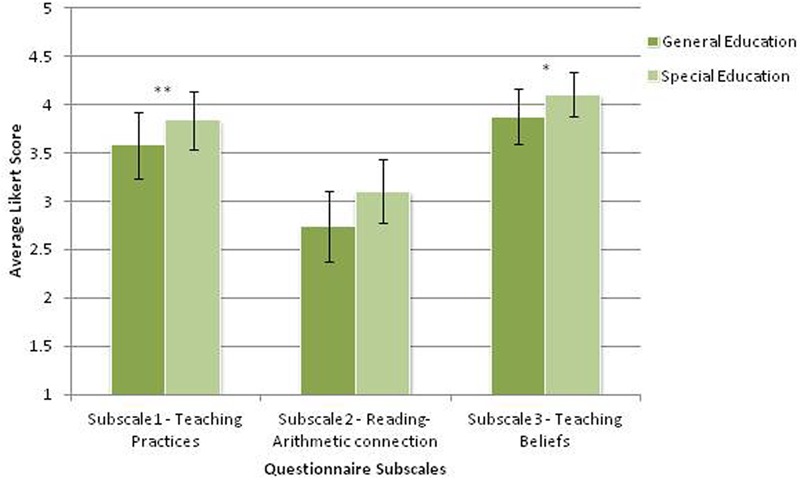
**Mean Iikert scores of questionnaire subscales, for general education and special education teachers.** Error bars represent standard errors. ^∗^*p* < 0.05, ^∗∗^*p* < 0.01.

It was found that special education teachers scored significantly higher on questionnaire subscales 1 (TP) and 3 (TB) compared to general education teachers. On subscale 2 (RAC), the difference was marginally significant. [Subscale 1. *F*(1,113) = 7.850, *p* < 0.01; Subscale 2. *F*(1,113) = 3.673, *p* = 0.058; Subscale 3. *F*(1,113) = 5.042, *p* < 0.05; Wilk’s Λ = 0.911, see **Table 6** and **Figure 1**).

**Table 6:** Multivariate analysis of covariance by “education system.”

Independent variables (IV)	General education		Special education		Dependent variables (DV)	Mean Square	*F* (df)	Partial eta squared
	*M*	*SD*	*M*	*SD*				
Education System	3.58	0.68	3.84	0.61	Subscale 1: Teaching practices (TP)	3.195	7.850 (1,113)^∗∗^	0.065
	2.74	0.81	3.10	0.76	Subscale 2: Reading-arithmetic connection (RAC)	2.260	3.673 (1,113)ˆ	0.031
	3.88	0.56	4.11	0.45	Subscale 3: Teaching beliefs (TB)	1.366	5.042 (1,113)^∗^	0.043
Covariate (years Of experience)					Subscale 1	2.822	6.934 (1,113)^∗^	
					Subscale 2	1.297	2.108 (1,113)	
					Subscale 3	0.009	0.032 (1,113)	

*^∗^*p* < 0.05, ^∗∗^*p* < 0.01, ^p = 0.058, N = 116.*

## Discussion

Findings from the current study add an important professional tool for teachers in the area of EF and academic achievement. First we present a new reliable and valid questionnaire that can be used to investigate beliefs of teachers and graduate students in schools of education, regarding the role of EF in reading and arithmetic class. In addition to assessing teachers’ current stand on this issue, the act of answering the questionnaire in itself can raise teacher awareness to the importance of EF for academic achievement. Furthermore, our results indicate that special and general education teachers see differently the contribution of EF to student competence in reading and arithmetic.

Mastery of EF processes such as goal setting, planning, organizing, prioritizing, memorizing, initiating, shifting, and self-monitoring are all essential for productive functioning in our progressively complex, technological society. In addition, EF has been a focus of the continuing theoretical debate concerning the origins of cognition and how it develops throughout life. Beginning in the elementary grades, students are asked to complete lengthy reading, writing and arithmetic assignments, all of which profoundly depend on these EF processes. Hence, academic as well as life success are thus dependent on students’ ability to plan their time, organize and prioritize materials and information, distinguish main idea from details, shift approaches flexibly, monitor their own progress, and reflect on their work. However, EF is not taught systematically in schools and is not a focus of the pedagogical curriculum (Meltzer et al., 2007). In addition, not many programs directly target how EF strategies are developed and implemented. There are a few published studies of such programs, such as “Tools of the mind” (Diamond et al., 2007) and PATHS (Promoting Alternative Thinking Strategies; Riggs, 2004). These studies mostly date back to the last decade and were conducted in North America (Dias and Seabra, 2015).

A strong indication of the neglect of EF in the educational setting, can be found in the guidelines of the National Reading and Math Panels of the United-States, which do not mention in any explicit form the role of underlying EF in the acquirement of reading (National Reading Panel, 2000) and math (National Mathematics Advisory Panel, 2008). The NRP does not address the role of EF in reading-comprehension, for example, or the need to educate teachers about domain-general cognitive variables affecting students’ literacy. Under these circumstances, it is of interest to investigate what teachers believe and do, in relation the contribution of EF to their students’ reading and arithmetic achievements. This is a question worth answering, since it is known that a change in practices can be achieved only if teachers’ attitudes and beliefs are addressed (Ertmer et al., 2012).

To address this issue, a novel questionnaire of 22 items was composed, divided into three subscales by an exploratory factoring procedure: “TP,” “RAC” and “TB” The questionnaire was found internally consistent, its content validity reviewed by professional and construct validity evaluated by the factoring process.

As hypothesized, a strong positive correlation (*r* = 0.512) emerged between respondents’ scores on the “TP” and “TB” questionnaire subscales, in agreement with the general notion that teaching beliefs are correlated with pedagogical practices (Baccus, 2004; Ertmer et al., 2006; Cross, 2009; Paro et al., 2009).

Scores on the “TB” and “RAC” subscales were also positively correlated, but to a lesser extent, suggesting that beliefs about the contribution of EF to reading and arithmetic are somewhat related to beliefs about a connection between reading and arithmetic abilities. The relative weakness of this correlation implies that these subscales do measure different sets of teaching beliefs. This conclusion is supported by a descriptive analysis of respondents’ scores on the different subscales, which suggests that most questionnaire respondents believe in a connection between executive functioning and achievements in reading and arithmetic (as measured by the TB subscale. *M* = 3.97 on a scale of 5). It may also suggest, to a lesser extent (due to higher variance in scores on the TP subscale), that most of them try addressing this connection in their classroom practices (*M* = 3.68). On the contrary, teachers vary greatly in the way they see the connection reading-arithmetic and the existence of shared underlying mechanisms, as measured by the RAC subscale (*M* = 2.88). Approximately half of them (49.14%) scored between 2.5 and 3.5 on this subscale, thus seem to be undecided on this issue, and about a third (31.03%) scored below 2.5, signaling their disagreement.

We can conclude from these findings, that most teachers recognize the effect that student behaviors reflecting cognitive flexibility, inhibition, attentional control, planning, working memory, and automatic retrieval, have on achievements in reading and arithmetic. Moreover, findings indicate that teachers’ beliefs about this effect are related to their reported teaching practices.

Furthermore, the fact that half of the teachers did not approve or deny the connection between the cognitive foundations of, and achievements in, reading and arithmetic actually supports the influence they attribute to underlying domain-general mechanisms. How so? Traditionally, cognitive mechanisms underlying reading and arithmetic are viewed as mostly domain-specific and separable (e.g., Lyon et al., 2003; Price et al., 2007). These mechanisms do play a central role, along with domain-general mechanisms as EF. Thus, agreement with statements in the RAC subscale (for example: “If a student has difficulty in both reading and math, these difficulties usually stem from the same source”) would deny the important part that phonological awareness does play in reading (Lyon et al., 2003) or the ability to manipulate quantities plays in arithmetic (Price et al., 2007). The “undecided” respondents actually signal in their responses that they believe reading and arithmetic rely on both domain-specific and domain-general mechanisms.

Our findings indicate that beliefs of professionals in the field are in agreement with the abundance of literature about the contribution of EF to reading and arithmetic abilities. However, teachers’ knowledge about EF remains intuitive. Without explicit training, teachers will continue to not identify EF as a contributor when a student experiences difficulties in reading or arithmetic. With a scarcity of evidence-based interventions addressing EF (Dias and Seabra, 2015), teachers will not be able to implement interventions suitable for students with EF difficulties. Furthermore, as research indicates, addressing EF in the classroom can help all students, not only struggling ones. As Meltzer et al. (2007) claimed, interventions addressing EF would result in less special-education referrals. It is time to include EF as an important factor in national reports, teacher training and school curriculum.

On another note, a negative correlation (*r* = -0.192) was found between score on the RAC subscale and overall years of experience, contrary to our hypotheses. We can conclude that the more years a person gained as a teacher, he/she tends to hold a more traditional view, of reading and arithmetic being supported mostly by domain-specific mechanisms. Experienced teachers were found in the past, to possess higher knowledge of characteristics of ADHD than inexperienced ones (Anderson et al., 2012), yet it does not necessarily mean they see EF as shared underlying mechanisms of reading and arithmetic abilities. To assess also whether our questionnaire differentiates between groups of teachers. We compared between general and special education teachers, controlling for years of experience, which served as a confounding variable. In compliance with our hypotheses, it was found that special education teachers scored significantly higher than general education teachers on the TP (*F* = 7.85, *p* < 0.01) and TB (*F* = 5.04, *p* < 0.05) questionnaire subscales. Thus, special education teachers reported holding more beliefs about the contribution of EF to achievements in reading and arithmetic, and also reported applying more teaching practices targeting EF in their reading and arithmetic classes. In addition, a marginally significant difference was found in scores on the RAC subscale (*F* = 3.67, *p* = 0.058), suggesting these teachers might also see a stronger connection between the cognitive foundations of, and achievements in, reading and arithmetic.

Although EF is known to affect achievements in reading and arithmetic in the general population (e.g., Altemeier et al., 2008; Miller et al., 2013), there are some reasons why this link may be less noticeable to the general education teacher. On the one hand, one of the central characteristics of the special education system is seeing students in a holistic way, starting by building an Individualized Education Program (IEP; Gartin and Murdick, 2005) for every student. This attention to every student’s overall functioning and thinking processes, makes the special education teacher more prone to notice domain-general mechanisms affecting achievements across different school subjects. On the other hand, another possible explanation relies in the fact that special education students do manifest more difficulties in EF (Wright, 2010). Teachers witnessing most of their students struggle greatly with basic executive functioning, such as inhibiting responses in class and shifting between simple tasks, may give more weight to the effect of these mechanisms on school achievement.

There are some limitations to this study which should be taken into account. First of all, the questionnaire developed, being the first of its kind, is still at its first steps in terms of validity and generalizability. In the future, the questionnaire should be administered to more groups of teachers, to enhance its value to the field. Moreover, the comparison made between general and special education teachers did produce interesting findings, but should be addressed carefully, since it was performed on the population used for questionnaire developed.

Lastly, there is no certainty that teaching practices reported are aligned with actual classroom practices. Naturally, they may reflect desirable, optimal classroom situations while actual pedagogical practices are affected by “classroom realities” (Ertmer et al., 2012). In general, questionnaire respondents tend to socially desirable responding, which is “the tendency of people to present a favorable image of themselves on questionnaires” (Van de Mortel, 2008). Yet, consequently, we can at least infer from the reported practices that teachers feel that addressing EF in reading and arithmetic classes would be “the right thing to do.” Additionally, the questionnaire, inquiring about such practices, has also an effect of raising teachers’ awareness, first to their beliefs about the contribution of EF, and secondly, to the way these beliefs align with their teaching practices.

In summary, the current study introduces a novel research questionnaire, investigating school teachers’ beliefs and practices concerning the contribution of EF to students’ achievements in reading and arithmetic. Our findings indicate that early elementary teachers hold beliefs about the contribution of EF to students’ reading and arithmetic achievements. Additionally, they report addressing this issue in their teaching practices. That is even more so in the case of special education teachers, compared to general education teachers. Further research, in more populations and methodologies, is required to expand our view on the way our school system sees the contribution of EF to academic achievements. It still is to be discovered how current research in this field can be communicated to teachers and manifested in the curriculum, and vice versa, how can teachers’ experience inform current research. By that, the bridge between researchers and teachers, science and the classroom, will continue to strengthen and thrive.

## Author Contributions

SR, OR, and TM contributed to the design of the work, its analysis, and interpretation of data. In addition, SR, OR, and TK drafted and wrote the work and gave final approval of the version to be published.

## Conflict of Interest Statement

The authors declare that the research was conducted in the absence of any commercial or financial relationships that could be construed as a potential conflict of interest.

The reviewer LC and the handling Editor declared their shared affiliation, and the handling Editor states that the process nevertheless met the standards of a fair and objective review.
