# Supplementary material for: Teachers’ Beliefs and Practices Regarding the Role of Executive Functions in Reading and Arithmetic
Source: Front Psychol. 2016 Oct 17;7:1567. doi: 10.3389/fpsyg.2016.01567 (PMC5065981; doi:10.3389/fpsyg.2016.01567)
Supplement: Supplementary file 1 [file Data_Sheet_1.docx]

**Appendices**

**Appendix 1. Collection of Demographic Data in Research Questionnaire**

**Education**: General / Special education (Circle the correct answer).

**חינוך**: רגיל / מיוחד (הקיפי את התשובה המתאימה)

**Age**: ___

**גיל**: ____

**Gender**: male / female (Circle the correct answer).

**מגדר**: ♀ אישה / ♂ איש (הקיפי את התשובה המתאימה)

**Education**: High-school / B.A. / M.A. (Circle the correct answer).

**השכלה**: תיכונית / תואר ראשון/ תואר שני (הקיפי את התשובה המתאימה)

If you were educated in an academic institute, what institute and what did you major in?

אם למדת במוסד אקדמי, באיזה מוסד ובאילו חוגי לימוד?

_________________________________________________________________________

**Teaching certificate:** Yes / No / Student (Circle the correct answer).

**תעודת הוראה:** כן / לא / לומדת לתעודת הוראה (הקיפי את התשובה המתאימה)

**Years of experience** teaching **reading** and language arts: ____

**שנות ניסיון** בהוראת **קריאה** וכישורי שפה: ____

How many **hours** **weekly** do you teach reading and language arts? ____

כמה **שעות שבועיות** את/ה מלמד/ת קריאה וכישורי שפה? ____

Which **textbooks** do you use for reading and language arts class? In which grade?

באילו **ספרי לימוד** אתם משתמשים בקריאה וכישורי שפה? באיזו כיתה?

________________________________________________________________________

**Years of experience** teaching **arithmetic**: ____

**שנות ניסיון** בהוראת **חשבון**: ____

How many **hours weekly** do you teach arithmetic? ____

כמה **שעות שבועיות** את/ה מלמד/ת חשבון? ____

Which **textbooks** do you use for arithmetic class? In which grade?

באילו **ספרי לימוד** אתם משתמשים בחשבון? באיזו כיתה?

________________________________________________________________________

Which grade/s do you teach? 1^st^ / 2^nd^ / 3^rd^ / 4^th^ / 5^th^ / 6^th^ (Circle the correct answers).

**אילו כיתות את/ה מלמד/ת**? א / ב / ג / ד / ה / ו (הקיפי את התשובות המתאימות)

If you do not teach 1^st^ or 2^nd^ grade, **how long ago did you teach these grades**? Which grade?

אם את/ה לא מורה בכיתה א' או ב', **לפני כמה זמן לימדת בכיתות אלו**? באיזו כיתה?

__________________________________________________________________________

**Homeroom** teacher **/** **professional** teacher / both. (Circle the correct answer).

**מחנכ/ת או מורה מקצועי/ת?** מחנכת / מורה מקצועית / גם וגם. (הקיפי את התשובה המתאימה).

**Appendix 2. Items of Research Questionnaire (in the order of appearance)**

The leftmost column states the questionnaire subscale the item belongs to (if any).

| Subscale | Mechanism | Item | Item in Hebrew |
| --- | --- | --- | --- |
|  | attention focusing | strategies for focusing on task should be taught at school | יש ללמד במסגרת בית הספר אסטרטגיות להתמקדות במשימה |
|  | attention focusing | in order to solve a math word problem, the student should understand the "big picture" and not only the details comprising it. | כדי לפתור בעיה מילולית בחשבון, יש להבין את "התמונה הרחבה" ולא רק את הפרטים המרכיבים אותה. |
|  | attention focusing | in order to solve a math problem, the student should understand the "big picture" and not only the details comprising it. | כדי לפתור תרגיל חשבוני, יש להבין את "התמונה הרחבה" ולא רק את הפרטים המרכיבים אותה. |
| 1. Teaching Practices | planning | I teach in math class strategies for planning ahead in task performance | אני מלמד/ת במסגרת שיעור חשבון אסטרטגיות לתכנון מראש בעת ביצוע מטלות |
| 1. Teaching Practices | working memory | when I teach a student with difficulties in math, I will work with him on methods to store in his memory multiple arithmetical operations in parallel | כשאני מלמד/ת בשעה פרטנית תלמיד המתקשה בחשבון, אעבוד איתו על שיטות לאחסן בזיכרון כמה פעולות חשבון במקביל. |
| 1. Teaching Practices | retrieval | I devote time in language arts class to memorizing **common** orthographic patterns, to encourage reading them as whole words instead of their phonological decoding | אני מקדיש/ה זמן בשיעור כישורי שפה לשינון של תבניות מילים שכיחות, על מנת לעודד את קריאתן באופן תבניתי ולא מצרף. |
|  | working memory | many times, students who find it difficult to remember complex instructions have also difficulties in math | פעמים רבות, תלמידים המתקשים לזכור הוראות מורכבות מתקשים גם בחשבון. |
|  | working memory | in order to solve math problems, one has to keep information about arithmetical operations in his memory | כדי לפתור תרגילים בחשבון, יש צורך לשמור מידע על פעולות חשבון בזיכרון. |
|  | flexibility | students who have difficulties in math also have difficulties in adjusting to tasks with new instructions | תלמידים המתקשים בחשבון גם מתקשים להסתגל למטלות עם הוראות חדשות. |
|  | inhibition | strategies supporting behavioral inhibition should be taught at school | יש ללמד במסגרת בית הספר אסטרטגיות לעיכוב תגובות |
| 3. Teaching Beliefs | planning | students who are able to plan ahead their actions in performing a task, cope better with math word problems. | לילדים שמסוגלים לתכנן מראש את הפעולות בהן ינקטו כדי לבצע מטלה, יותר קל להתמודד עם בעיות מילוליות בחשבון. |
| 1. Teaching Practices | attention focusing | I teach in language arts class strategies to focus on task | אני מלמד/ת במסגרת שיעור כישורי שפה אסטרטגיות להתמקדות במשימה |
|  | flexibility | students who have difficulties in reading comprehension, also have difficulties in adjusting to tasks demanding different strategies for solution | תלמידים המתקשים בהבנת הנקרא גם מתקשים להסתגל למטלות עם דרכי פתרון השונות מאלו של מטלות קודמות. |
| 2. Reading-Arithmetic correlation | Reading-Arithmetic correlation | the basic mechanisms crucial for learning math are also crucial for learning to read | המנגנונים הבסיסיים החיוניים ללמידת חשבון הם חיוניים גם ללמידת קריאה |
|  | flexibility | students who have difficulties in reading comprehension, also have difficulties in adjusting to tasks with new instructions | תלמידים המתקשים בהבנת הנקרא גם מתקשים להסתגל למטלות עם הוראות חדשות. |
| 2. Reading-Arithmetic correlation | Reading-Arithmetic correlation | most of the children who read well are also good in math | רוב הילדים שקוראים היטב טובים גם בחשבון. |
| 2. Reading-Arithmetic correlation | Reading-Arithmetic correlation | there are more students who have difficulties both in reading and math, than students with difficulties in reading only and not in math | יש יותר תלמידים המתקשים גם בקריאה וגם בחשבון מאשר תלמידים המתקשים רק בקריאה אבל לא בחשבון. |
|  | phonological awareness | I devote time in language arts class to practice reading unfamiliar words by breaking them into sounds | אני מקדיש זמן בשיעור כישורי שפה לתרגול קריאת מילים לא-מוכרות בעזרת פירוק לצלילים. |
|  | attention focusing | I teach in math class strategies for focusing on task | אני מלמד/ת במסגרת שיעור חשבון אסטרטגיות להתמקדות במשימה |
|  | phonological awareness | students who are good in math are also good in breaking a word into its sounds | תלמידים שטובים בחשבון טובים גם בפירוק מילה לצלילים. |
|  | inhibition | I teach in language arts class strategies supporting behavioral inhibition | אני מלמד/ת במסגרת שיעור כישורי שפה אסטרטגיות לעיכוב תגובות |
|  | flexibility | flexibility when switching between tasks should be taught at school | יש ללמד במסגרת בית הספר אימון בגמישות במקרים של מעבר בין מטלות |
| 3. Teaching Beliefs | attention focusing | the ability to focus on task is important when solving math problems | היכולת להתמקד במשימות חשובה לפתרון תרגילים בחשבון |
|  | general verbal ability | the general verbal ability (vocabulary, syntax, morphology) affects the acquisition of reading | היכולת השפתית הכללית (אוצר מילים, תחביר, מורפולוגיה) משפיעה על רכישת הקריאה. |
|  | general verbal ability | children with a small vocabulary will have difficulty in the acquisition of math | ילדים עם אוצר מילים מצומצם יתקשו ברכישת החשבון. |
| 3. Teaching Beliefs | flexibility | students with difficulties in reading comprehension also tend to try solving problems again and again in the same way, even if this way was proven wrong | תלמידים המתקשים בהבנת הנקרא גם נוטים לנסות לפתור בעיות שוב ושוב באותה דרך גם אם דרך זו הוכחה כלא-נכונה. |
|  | retrieval | a difficulty in math stems from inability to recall the solution of a math problem previously calculated by the student | קושי בחשבון נובע מחוסר יכולת להיזכר בתוצאה של תרגיל שחושב על ידי התלמיד/ה בעבר. |
|  | Reading-Arithmetic correlation | students with reading problems should be supported in the process of acquiring math | יש לתמוך בתלמידים עם בעיות קריאה בתהליך למידת החשבון. |
| 1. Teaching Practices | working memory | when i teach a student with difficulties in reading, I will work with him on methods to store in his memory multiple bits of information in parallel | כשאני מלמד/ת בשעה פרטנית תלמיד המתקשה בקריאה, אעבוד איתו על שיטות לאחסון בזיכרון של כמה פרטי מידע במקביל. |
|  | planning | children who can plan ahead their actions in performing a task, perform better in reading comprehension | לילדים שמסוגלים לתכנן מראש את הפעולות בהן ינקטו כדי לבצע מטלה, יותר קל להבין את הנקרא. |
|  | retrieval | to achieve progress in reading, the student should be able to read globally, rather than decode, words he has seen many times before | כדי להתקדם בקריאה, על התלמיד לקרוא מילים שראה פעמים רבות בעבר לפי תבנית המילה במקום בקריאה מצרפת. |
| 3. Teaching Beliefs | attention focusing | the ability to focus on task is important for reading comprehension | היכולת להתמקד במשימות חשובה להבנת הנקרא. |
| 3. Teaching Beliefs | working memory | one has to keep in memory information while reading, in order to achieve reading comprehension | כדי להבין את הנקרא, יש צורך לשמור בזיכרון מידע במהלך הקריאה. |
|  | inhibition | inhibition is an important ability in the acquirement of math | היכולת לעכב תגובות חשובה לרכישת החשבון. |
|  | attention focusing | in order to achieve reading comprehension, one should understand the "big picture" and not only the details comprising it. | כדי להבין את הנקרא, יש להבין את "התמונה הרחבה" ולא רק את הפרטים המרכיבים אותה. |
| 2. Reading-Arithmetic correlation | Reading-Arithmetic correlation | there are more students who have difficulties both in reading and math, than students with difficulties in math only and not in reading | יש יותר תלמידים המתקשים גם בקריאה וגם בחשבון מאשר תלמידים המתקשים רק בחשבון אבל לא בקריאה. |
| 2. Reading-Arithmetic correlation | Reading-Arithmetic correlation | students who do not read accurately have difficulties in understanding math | תלמידים המתקשים לדייק בקריאה מתקשים גם בהבנה חשבונית. |
|  | planning | I teach in language arts class strategies for planning ahead in task performance | אני מלמד/ת במסגרת שיעור כישורי שפה אסטרטגיות לתכנון מראש בעת ביצוע מטלות |
| 3. Teaching Beliefs | flexibility | students with difficulties in math also tend to try solving problems again and again in the same way, even if this way was proven wrong | תלמידים המתקשים בחשבון גם נוטים לנסות לפתור בעיות שוב ושוב באותה דרך גם אם דרך זו הוכחה כלא-נכונה. |
|  | working memory | I teach in math class strategies to remember multiple arithmetical operations in parallel | במסגרת שיעור חשבון, אני מלמד/ת אסטרטגיות לזכירה של כמה פעולות חשבון בו-זמנית |
| 3. Teaching Beliefs | retrieval | the student's ability to quickly recall the spelling of words he has previously been exposed to, affects reading rate | היכולת של תלמיד להיזכר מהר בדרך בה נכתבות מילים שנחשף אליהן בעבר משפיעה על מהירות הקריאה. |
| 3. Teaching Beliefs | planning | children who can plan ahead their actions in performing a task, solve math problems more easily | לילדים שמסוגלים לתכנן מראש את הפעולות בהן ינקטו כדי לבצע מטלה, יותר קל לפתור תרגילים בחשבון. |
|  | Reading-Arithmetic correlation | if a student has difficulty in both reading and math, these difficulties usually stem from two different sources | אם תלמיד מתקשה גם בקריאה וגם בחשבון, בדרך כלל הקשיים נובעים משני מקורות שונים. |
|  | flexibility | students who have difficulties in math, also have difficulties in adjusting to tasks demanding different strategies for solution | תלמידים המתקשים בחשבון גם מתקשים להסתגל למטלות עם דרכי פתרון השונות מאלו של מטלות קודמות. |
|  | phonological awareness | student who can break a word into its sounds are usually good readers | תלמידים שיודעים לפרק מילה לצליליה הם בדרך כלל קוראים טובים. |
|  | Reading-Arithmetic correlation | the basic mechanisms crucial for learning to read are also crucial for learning math | המנגנונים הבסיסיים החיוניים ללמידת קריאה הם חיוניים גם ללמידת חשבון |
|  | attention focusing | children who are good in math also show a high ability to focus on tasks | ילדים שטובים בחשבון מראים גם יכולת גבוהה להתמקד במשימות |
| 3. Teaching Beliefs | inhibition | inhibition is an important ability in the acquirement of reading | היכולת לעכב תגובות חשובה לרכישת הקריאה. |
|  | inhibition | I teach in math class strategies supporting behavioral inhibition | אני מלמד/ת במסגרת שיעור חשבון אסטרטגיות לעיכוב תגובות |
|  | flexibility | I teach, in language arts class, flexibility when switching between tasks | אני מלמד/ת במסגרת שיעור כישורי שפה אימון בגמישות במקרים של מעבר בין מטלות |
|  | retrieval | to achieve progress in math, the student should be able to recall the solutions of math problems he has been exposed to many times before, instead of recalculating them | כדי להתקדם בחשבון, על התלמיד להיזכר בתוצאות של תרגילים שראה פעמים רבות בעבר, במקום לחשב את התוצאה בכל פעם. |
| 2. Reading-Arithmetic correlation | Reading-Arithmetic correlation | if a student has difficulty in both reading and math, these difficulties usually stem from the same source | אם תלמיד מתקשה גם בקריאה וגם בחשבון, בדרך כלל הקשיים נובעים מאותו מקור. |
|  | planning | strategies for planning ahead in task performance should be taught at school | יש ללמד במסגרת בית הספר אסטרטגיות לתכנון מראש בעת ביצוע מטלות |
|  | retrieval | children with difficulties in retrieval struggle also with math | ילדים עם קשיים בשליפה מתקשים בחשבון. |
|  | retrieval | the student's ability to quickly recall the solutions to problems he has previously been exposed to, affects performance in math | היכולת של תלמיד להיזכר מהר בפתרונות של תרגילים שנחשף אליהם בעבר משפיעה על הביצועים בחשבון. |
|  | working memory | in order to solve math word problems, one has to keep information about arithmetical operations in his memory | כדי להבין בעיות מילוליות בחשבון, יש צורך לשמור מידע על פעולות חשבון בזיכרון. |
|  | phonological awareness | one has to master the rules of phonological decoding, in order to achieve reading comprehension | כדי להבין את הנקרא, התלמיד צריך לדעת את כל חוקי הפענוח הפונולוגיים. |
|  | inhibition | many times, I have witnessed students with difficulties in inhibition, having difficulties in math | ראיתי במקרים רבים קשיים בחשבון אצל תלמידים המתקשים לעכב תגובות. |
|  | Reading-Arithmetic correlation | most of the children who are good in math also read well | רוב הילדים שטובים בחשבון גם קוראים היטב. |
|  | attention focusing | children with good reading comprehension also show a high ability to focus on tasks | ילדים שטובים בהבנת הנקרא מראים גם יכולת גבוהה להתמקדות במשימות |
| 1. Teaching Practices | working memory | I teach, in language arts class, strategies to remember in parallel multiple details from the text | במסגרת שיעור כישורי שפה, אני מלמד/ת אסטרטגיות לזכירה של מספר פרטים מהטקסט במקביל |
|  | general verbal ability | the general verbal ability (vocabulary, syntax, morphology) affects the acquisition of math | היכולת השפתית הכללית (אוצר מילים, תחביר מורפולוגיה) משפיעה על רכישת החשבון. |
|  | retrieval | children with difficulties in retrieval struggle also with reading | ילדים עם קשיים בשליפה מתקשים בקריאה. |
|  | working memory | many times, students who find it difficult to remember complex instructions have also difficulties in reading | פעמים רבות, תלמידים המתקשים לזכור הוראות מורכבות מתקשים גם בקריאה. |
| 1. Teaching Practices | retrieval | I devote time in math class to memorizing solutions to common math problems | אני מקדיש/ה זמן בשיעור חשבון לשינון של פתרונות לתרגילים שכיחים ואחסונם בזיכרון. |
|  | general verbal ability | students with language problems should be supported in the process of reading acquirement | יש לתמוך בתלמידים עם בעיות שפה כבר מתחילת תהליך למידת הקריאה. |
|  | flexibility | I teach, in math class, flexibility when switching between tasks | אני מלמד/ת במסגרת שיעור חשבון גמישות במקרים של מעבר בין מטלות |
|  | inhibition | many times, I have witnessed students with difficulties in inhibition, having difficulties in math | ראיתי במקרים רבים קשיים בהבנת הנקרא אצל תלמידים המתקשים לעכב תגובות. |
|  | general verbal ability | children with a small vocabulary will have difficulty in the acquisition of reading | ילדים עם אוצר מילים מצומצם יתקשו ברכישת הקריאה. |
